# Supplementary material for: Relationship Between Body Composition and Biomarkers in Adult Females with Breast Cancer: 1-Year Follow-Up Prospective Study
Source: Nutrients. 2025 Jul 30;17(15):2487. doi: 10.3390/nu17152487 (PMC12348593; doi:10.3390/nu17152487)
Supplement: Supplementary file 1 [file nutrients-17-02487-s001.zip › nutrients-3763629-supplementary.pdf]

Supplementary table S1: Bivariate correlation matrix for absolute changes between body composition analysis, strength, and biomarkers

	ΔGlyc (mg/dl)	ΔHbA1c (%)	ΔInsulin (µU/mL)	ΔHOMA-IR	ΔTotal-cholesterol (mg/dL)	ΔHDL-cholesterol (mg/dL)	Δnon-HDL-cholesterol (mg/dL)	ΔLDL-cholesterol (mg/dL)	ΔTriglycerides (mg/dL)	ΔCRP (mg/L)	ΔIL-6 (pg/mL)	ΔVitamin D (ng/mL)	ΔMyostatin (ng/mL)	ΔFGF-21 (pg/mL)
**Anthropometry**														
**ΔWeight (kg)**	,158	-,179	,264	,301	,136	**-,319**	,206	,142	,246	,245	,195	**-,344**	**-,362**	-,181
**p**	,228	,228	,067	,070	,317	**,017**	,131	,300	,068	,083	,170	**,012**	**,020**	,265
**ΔBMI (kg/m²)**	,149	-,174	,247	,272	,139	**-,311**	,209	,138	**,270**	,250	,205	**-,311**	**-,361**	-,172
**p**	,256	,241	,087	,104	,308	**,021**	,126	,313	**,044**	,077	,149	**,024**	**,020**	,288
**ΔWC (cm)**	,132	,040	-,079	-,015	-,028	-,243	,046	,015	,054	,090	**-,325**	-,045	-,111	,183
**p**	,315	,791	,588	,928	,835	,074	,738	,915	,690	,530	**,020**	,748	,490	,259
**ΔWC/Ht**	,126	,033	-,083	-,025	-,028	-,244	,046	,012	,059	,087	**-,319**	-,042	-,109	,182
**p**	,336	,824	,572	,885	,836	,073	,740	,929	,666	,542	**,023**	,763	,499	,261
**Electrical and water-derived BIA analysis**														
**ΔRz (Ω)**	,035	,070	,006	,206	,246	-,069	,247	,201	,208	,143	,118	,161	-,093	-,086
**p**	,791	,642	,968	,221	,068	,616	,069	,141	,125	,317	,409	,250	,565	,596
**ΔXc (Ω)**	,029	,095	,134	,265	,142	-,221	,192	,116	,258	,133	,059	-,014	-,104	-,137
**p**	,824	,526	,359	,112	,296	,106	,160	,399	,055	,352	,678	,920	,519	,401
**ΔPhA (˚)**	-,130	,149	,086	,123	,005	-,215	,081	,013	,135	,115	-,057	-,045	,000	-,145
**p**	,322	,316	,555	,468	,971	,115	,559	,926	,320	,423	,693	,751	1,000	,372
**ΔPhA/BMI (˚/kg*m²)**	-,106	**,378**	-,066	-,111	-,080	,182	-,115	-,108	-,132	-,138	-,185	,198	,163	,034
**p**	,420	**,009**	,650	,514	,558	,185	,402	,434	,332	,333	,195	,155	,308	,834
**ΔECW/TBW (%)**	-,027	**-,303**	-,216	-,243	,006	,225	-,084	,000	-,191	-,111	,129	,112	-,044	,051
**p**	,837	**,038**	,141	,153	,968	,103	,547	1,000	,162	,444	,370	,430	,788	,757
**Fat-related parameters (BIA and US)**														
**ΔFM (%)**	,167	-,266	**,330**	**,522**	,180	-,252	,233	,152	,218	,260	,268	-,037	-,069	-,097
**p**	,203	,071	**,021**	**,001**	,184	,063	,086	,269	,107	,065	,058	,793	,669	,552
**ΔFM (kg)**	,192	-,253	**,318**	**,463**	,188	-,237	,243	,167	,242	,264	,249	-,170	-,228	-,132
**p**	,141	,087	**,026**	**,004**	,166	,081	,074	,222	,072	,061	,078	,224	,151	,415
**ΔFMI (kg/m²)**	,205	-,237	**,336**	**,481**	,163	-,241	,218	,137	,235	,243	,239	-,127	-,219	-,114
**p**	,116	,109	**,018**	**,003**	,229	,076	,109	,319	,082	,086	,091	,366	,170	,485
**ΔVF (L)**	,135	,031	-,029	,034	,122	-,206	,176	,090	,257	**,308**	,020	,012	,102	,175
**p**	,315	,844	,847	,849	,383	,144	,211	,525	,063	**,033**	,893	,935	,543	,300
**ΔThigh SAT (cm)**	,040	,036	**,348**	,175	,021	-,185	,061	,014	,090	-,029	,053	-,108	,128	,060
**p**	,776	,826	**,026**	,346	,888	,214	,686	,928	,542	,855	,735	,482	,449	,726
**ΔtASAT (cm)**	,128	-,041	,076	-,001	-,024	**-,444**	,086	-,063	**,377**	-,055	,175	-,172	-,007	-,176
**p**	,366	,802	,634	,994	,871	**,002**	,564	,676	**,008**	,723	,255	,257	,968	,292
**ΔsASAT (cm)**	,261	-,151	,300	,222	,007	**-,407**	,053	-,095	**,412**	,024	**,337**	-,002	,158	-,152
**p**	,061	,353	,054	,221	,963	**,004**	,725	,527	**,004**	,875	**,025**	,992	,344	,362
**ΔdASAT (cm)**	-,076	,026	-,262	-,322	-,075	-,270	,043	-,042	,185	-,139	-,064	-,153	-,121	-,083
**p**	,093	,073	,615	,067	,774	,780	,208	,368	,679	,093	,073	,315	,470	,618
**ΔPreperitonealAT (cm)**	-,030	-,147	,267	,230	,013	-,168	,106	,117	,019	,166	,073	**-,300**	-,253	,026
**p**	,833	,365	,087	,205	,930	,259	,478	,434	,898	,283	,639	**,045**	,125	,875
**Fat free mass and muscle-related parameters (BIA and US)**														
**ΔFFMI (kg/m²)**	-,175	,006	-,136	**-,327**	-,166	-,193	-,095	-,083	-,065	-,114	-,066	-,221	-,237	-,102

**p**	,180	,966	,351	**,049**	,222	,157	,489	,549	,634	,426	,646	,111	,135	,533
**ΔSMMI (kg/m²)**	-,041	-,027	-,018	-,211	**-,267**	,076	**-,267**	-,221	-,207	-,124	-,074	-,158	,052	,026
**p**	,755	,855	,903	,209	**,047**	,580	**,049**	,105	,125	,386	,606	,259	,745	,871
**ΔASMMI (kg/m²)**	,134	-,086	,212	,143	,074	**-,282**	,139	,072	,203	,165	,112	**-,306**	-,213	-,192
**p**	,306	,567	,143	,397	,585	**,037**	,312	,600	,133	,247	,432	**,026**	,181	,235
**Δ RF thickness (cm)**	-,146	-,083	-,053	,110	-,017	-,049	,036	,028	-,103	-,267	-,122	,073	**-,329**	-,032
**p**	,303	,609	,742	,554	,911	,745	,810	,851	,487	,084	,437	,635	**,047**	,850
**ΔRF-CSA (cm²)**	-,101	,080	,006	-,114	,043	,208	,075	,012	-,038	-,304	-,037	,284	-,267	-,092
**p**	,482	,628	,969	,549	,775	,166	,621	,938	,797	,051	,817	,062	,116	,594
**Fat free mass and muscle-derived parameters after correction for fat mass-derived parameters or vice versa (BIA)**														
**ΔFM/FFM ratio**	,226	-,238	**,358**	**,538**	,216	-,219	,261	,179	,249	,257	,245	-,078	-,068	-,061
**p**	,082	,107	**,012**	**,001**	,110	,109	,054	,191	,065	,069	,083	,579	,675	,708
**ΔSMM/weight (%)**	-,179	**,407**	-,163	**-,351**	**-,269**	,006	-,237	-,214	-,106	-,117	-,225	-,090	,031	-,068
**p**	,172	**,005**	,263	**,033**	**,045**	,966	,082	,117	,437	,416	,112	,522	,846	,679
**ΔASMM/BMI (kg/kg*m²)**	-,066	,241	-,145	-,250	-,208	**,289**	**-,283**	-,223	-,250	**-,350**	-,242	,156	,265	,105
**p**	,616	,103	,319	,135	,125	**,033**	**,036**	,102	,063	**,012**	,088	,264	,094	,520
**Muscle Strength**														
**ΔHGS (kg)**	-,175	,093	-,126	-,255	,155	-,014	,147	,211	,007	-,045	-,206	,085	-,069	-,113
**p**	,181	,532	,390	,127	,253	,921	,285	,121	,957	,755	,148	,545	,669	,486
**ΔHGS/SMM ratio**	-,155	,055	-,203	-,234	,177	,122	,105	,186	-,052	-,111	-,160	,260	,011	-,011
**p**	,237	,712	,162	,164	,193	,376	,445	,175	,702	,437	,262	,060	,946	,946

The first row of each variable shows rho-Spearman correlation.

ASMM: Appendicular Skeletal Muscle Mass; ASMMI: Appendicular Skeletal Muscle Mass Index; AT: Adipose Tissue; BIA: bioelectrical impedance analysis; BMI: Body Mass Index; CRP: C-reactive protein; dASAT: deep abdominal sub-cutaneous adipose tissue; ECW: Extracellular Water; FGF-21: Fibroblast growth factor 21; FFM: Fat-Free Mass; FFMI: Fat-Free Mass Index; FM: Fat Mass; FMI: Fat Mass Index; Glyc: Glycemia; HbA1c: glycosylated haemoglobin; HDL: High-density lipoprotein; HGS: Handgrip strength; HOMA-IR: Insulin Resistance Homeostatic Model Assessment; IL-6: Interleukin 6; LDL: Low density lipoproteins; PhA: Phase Angle; RF: rectus femoris; RF-CSA: rectus femoris cross-sectional area; Rz: Resistance; SAT: subcutaneous adipose tissue; sASAT: superficial abdominal subcutaneous adipose tissue; SMM: Skeletal Muscle Mass; SMMI: Skeletal Muscle Mass index; tASAT: total abdominal subcutaneous adipose tissue; TBW: Total Body Water; US: ultrasound; VF: Visceral Fat; WC: Waist Circumference; WHtR: Waist–to–Height Ratio; Xc: Reactance.

Supplementary table S2: Bivariate correlation matrix for relative changes between body composition analysis, strength, and biomarkers

	Δ%Glyc	Δ%HbA1c	Δ%Insulin	Δ%HOMA	Δ%Total-cholesterol	Δ%HDL-cholesterol	Δ%non-HDL-cholesterol	Δ%LDL-cholesterol	Δ% Triglycerides	Δ%CRP	Δ%IL-6	Δ%Vitamin D	Δ%Myostatin	Δ%FGF-21
**Anthropometry**														
**Δ%Weight**	,152	-,160	,238	,238	,151	**-,336**	,197	,132	,249	,276	,191	**-,377**	**-,350**	-,208
**p**	,246	,282	,100	,156	,268	**,012**	,148	,336	,065	,050	,180	**,005**	**,025**	,198
**Δ%BMI**	,133	-,175	,223	,225	,163	**-,332**	,211	,141	**,269**	,272	,197	**-,333**	**-,360**	-,220
**p**	,309	,239	,124	,180	,230	**,013**	,121	,303	**,045**	,054	,166	**,015**	**,021**	,173
**Δ%WC**	,140	,026	-,037	-,071	-,024	-,247	,035	,035	,082	,091	**-,327**	-,054	-,139	,172
**p**	,285	,863	,802	,675	,861	,069	,798	,801	,548	,527	**,019**	,700	,388	,288
**Δ%WC/Ht**	,140	,026	-,037	-,071	-,024	-,247	,035	,035	,082	,091	**-,327**	-,054	-,139	,172
**p**	,285	,863	,802	,675	,861	,069	,798	,801	,548	,527	**,019**	,700	,388	,288
**Electrical and water-derived BIA analysis**														
**Δ%Rz**	,016	-,035	,001	,165	,245	-,082	,234	,186	,228	,134	,086	,159	-,089	-,106
**p**	,904	,815	,994	,330	,069	,554	,085	,174	,091	,347	,549	,257	,581	,515
**Δ%Xc**	,012	,066	,120	,192	,143	-,249	,182	,101	**,286**	,140	,014	,028	-,049	-,221
**p**	,929	,658	,413	,256	,293	,067	,184	,463	**,033**	,328	,921	,844	,759	,171
**Δ%PhA**	-,162	,128	,017	,050	,007	-,248	,083	,024	,149	,090	-,096	-,015	,021	-,231
**p**	,217	,391	,909	,768	,962	,068	,545	,861	,272	,529	,504	,917	,898	,152
**Δ%PhA/BMI**	-,148	**,391**	-,146	-,185	-,099	,128	-,110	-,101	-,084	-,085	-,202	,197	,160	-,036
**p**	,259	**,007**	,315	,274	,466	,353	,424	,462	,537	,555	,155	,157	,318	,824
**Δ%ECW/TBW**	-,044	-,256	-,261	-,243	,014	,230	-,063	,025	-,188	-,096	,143	,093	-,100	,135
**p**	,742	,082	,073	,153	,918	,095	,653	,858	,170	,506	,321	,510	,539	,413
**Fat-related parameters (BIA and US)**														
**Δ%FM_%**	,128	**-,317**	**,283**	**,466**	,176	**-,285**	,199	,105	,200	,262	,264	-,054	-,056	-,159
**pp**	,331	**,030**	**,048**	**,004**	,195	**,035**	,146	,444	,140	,063	,061	,699	,728	,326
**Δ%FM_kg**	,138	**-,288**	,236	**,368**	,175	**-,297**	,210	,120	,229	**,308**	,253	-,185	-,176	-,209
**p**	,294	**,049**	,102	**,025**	,197	**,028**	,124	,385	,090	**,028**	,074	,185	,270	,196
**Δ%FMI**	,144	-,279	,248	**,391**	,160	**-,298**	,193	,100	,231	**,285**	,253	-,162	-,165	-,206
**p**	,271	,058	,086	**,017**	,240	**,027**	,159	,468	,087	**,042**	,073	,246	,304	,202
**Δ%VF**	,135	-,019	,019	,077	,090	-,212	,127	,054	,179	,224	-,036	,047	,101	,169
**p**	,316	,901	,900	,664	,521	,131	,368	,705	,201	,126	,810	,748	,545	,319
**Δ%Thigh SAT**	,007	-,106	**,351**	,224	-,054	-,252	-,023	-,057	,069	,091	,088	-,229	,131	-,120
**p**	,961	,515	**,024**	,226	,716	,087	,876	,702	,641	,562	,576	,131	,439	,478
**Δ%tASAT**	,152	-,076	,162	,067	-,051	**-,461**	,030	-,109	**,319**	-,011	,114	-,222	,031	-,212
**p**	,283	,641	,304	,717	,731	**,001**	,842	,464	**,027**	,945	,460	,142	,854	,200
**Δ%sASAT**	,246	-,249	,264	,208	-,021	**-,414**	-,002	-,126	**,368**	,014	,226	-,056	,178	-,187
**p**	,078	,122	,091	,254	,887	**,004**	,990	,399	**,010**	,927	,140	,717	,285	,260
**Δ%dASAT**	-,110	-,006	-,096	-,230	-,039	-,245	,062	-,049	,205	-,152	-,024	-,199	-,131	-,225
**p**	,439	,970	,544	,205	,791	,097	,681	,743	,163	,326	,879	,191	,435	,174
**Δ%PreperitonealAT**	-,048	-,181	,263	,218	,045	-,237	,106	,182	,079	,168	,067	**-,295**	-,164	-,057
**p**	,736	,264	,092	,230	,764	,109	,480	,220	,595	,275	,663	**,049**	,325	,733
**Fat free mass and muscle-related parameters (BIA and US)**														

**Δ%FFMI**	-,175	,055	-,097	**-,363**	-,130	-,178	-,068	-,013	,011	-,109	-,064	-,267	-,229	-,152
**p**	,181	,714	,507	**,027**	,339	,194	,624	,922	,935	,448	,653	,053	,150	,348
**Δ%SMMI**	-,020	,051	,001	-,149	-,260	,099	-,256	-,207	-,237	-,147	-,075	-,132	,094	,092
**p**	,880	,733	,996	,378	,053	,471	,059	,129	,079	,303	,602	,346	,559	,573
**Δ%ASMMI**	,135	-,074	,218	,121	,103	**-,280**	,155	,094	,222	,166	,110	**-,341**	-,204	-,240
**p**	,304	,620	,131	,477	,449	**,039**	,259	,494	,100	,244	,441	**,012**	,202	,136
**Δ %RF thickness**	-,170	-,087	-,081	,050	,038	,016	,083	,117	-,058	-,263	-,049	,036	-,251	-,050
**p**	,227	,593	,616	,790	,797	,917	,580	,434	,697	,089	,756	,817	,134	,769
**Δ%RF-CSA**	-,118	,047	-,164	-,291	,106	,290	,148	,088	,016	**-,329**	,047	**,314**	-,291	-,189
**p**	,411	,776	,313	,118	,479	,050	,328	,560	,916	**,033**	,769	**,038**	,086	,270
**Fat free mass and muscle-derived parameters after correction for fat mass-derived parameters or vice versa (BIA)**														
**Δ%FM/FFM ratio**	,170	**-,301**	**,287**	**,480**	,208	-,247	,229	,137	,213	,268	,240	-,063	-,069	-,129
**p**	,193	**,040**	**,046**	**,003**	,123	,069	,093	,320	,115	,058	,089	,654	,669	,429
**Δ%SMM/weight**	-,206	**,457**	-,150	**-,391**	-,259	,026	-,219	-,182	-,067	-,103	-,217	-,095	,039	-,055
**p**	,114	**,001**	,302	**,017**	,054	,848	,108	,182	,623	,472	,127	,500	,808	,735
**Δ%ASMM/BMI**	-,073	**,301**	-,184	-,259	-,226	**,303**	**-,268**	-,202	-,232	**-,337**	-,213	,159	,245	,111
**p**	,577	**,040**	,207	,122	,094	**,025**	**,048**	,139	,085	**,016**	,134	,257	,122	,496
**Muscle Strength**														
**Δ%HGS**	-,182	,123	-,091	-,268	,156	-,021	,162	,234	-,015	-,041	-,216	,067	-,116	-,117
**p**	,164	,409	,533	,109	,251	,880	,238	,086	,914	,776	,127	,634	,470	,472
**Δ%HGS/SMM ratio**	-,155	,053	-,188	-,257	,173	,124	,118	,194	-,066	-,102	-,160	,259	-,026	-,005
**p**	,236	,724	,196	,124	,203	,367	,389	,157	,628	,477	,261	,061	,871	,973

The first row of each variable shows rho-Spearman correlation.

ASMM: Appendicular Skeletal Muscle Mass; ASMMI: Appendicular Skeletal Muscle Mass Index; AT: Adipose Tissue; BIA: bioelectrical impedance analysis; BMI: Body Mass Index; CRP: C-reactive protein; dASAT: deep abdominal sub-cutaneous adipose tissue; ECW: Extracellular Water; FGF-21: Fibroblast growth factor 21; FFM: Fat-Free Mass; FFMI: Fat-Free Mass Index; FM: Fat Mass; FMI: Fat Mass Index; Glyc: Glycemia; HbA1c: glycosylated haemoglobin; HDL: High-density lipoprotein; HGS: Handgrip strength; HOMA-IR: Insulin Resistance Homeostatic Model Assessment; IL-6: Interleukin 6; LDL: Low density lipoproteins; PhA: Phase Angle; RF: rectus femoris; RF-CSA: rectus femoris cross-sectional area; Rz: Resistance; SAT: subcutaneous adipose tissue; sASAT: superficial abdominal subcutaneous adipose tissue; SMM: Skeletal Muscle Mass; SMMI: Skeletal Muscle Mass index; tASAT: total abdominal subcutaneous adipose tissue; TBW: Total Body Water; US: ultrasound; VF: Visceral Fat; WC: Waist Circumference; WHtR: Waist–to–Height Ratio; Xc: Reactance.

Supplementary table S3: Multiple linear regression models used to explain the relationship between changes in body composition parameters (independent variable) and the evolution of biomarkers (dependent variable): crude models and adjusted for age.

Associated rank-transformed variables	Standardized β; p
ΔWeight - ΔHDL-cholesterol	Crude: -0.319; 0.018<br>Adjusted: -0.298; 0.030
ΔWeight - ΔVitamin D	Crude: -0.339; 0.013<br>Adjusted: -0.356; 0.011
ΔWeight - ΔMyostatin	Crude: -0.360; 0.021<br>Adjusted: -0.348; 0.027
ΔBMI - ΔHDL-cholesterol	Crude: -0.289; 0.034<br>Adjusted: -0.296; 0.030
ΔBMI - ΔTriglycerides	Crude: 0.272; 0.043<br>Adjusted: 0.302; 0.028
ΔBMI - ΔVitamin D	Crude: -0.308; 0.025<br>Adjusted: -0.322; 0.022
ΔBMI - ΔMyostatin	Crude: -0.360; 0.021<br>Adjusted: -0.348; 0.027
ΔWC -ΔIL-6	Crude: -0.322; 0.021<br>Adjusted: -0.318; 0.024
ΔWC/Ht-ΔIL-6	Crude: -0.317; 0.024<br>Adjusted: -0.313; 0.027
ΔPhA/BMI - ΔHbA1c	Crude: 0.386; 0.007<br>Adjusted: 0.415; 0.004
ΔECW/TBW -ΔHbA1c	Crude: -0.311; 0.033<br>Adjusted: -0.314; 0.032
ΔFM -ΔInsulin	Crude: 0.330; 0.020<br>Adjusted: 0.303; 0.039
ΔFM - ΔHOMA-IR	Crude: 0.503; 0.002<br>Adjusted: 0.466; 0.006
ΔFM - ΔInsulin	Crude: 0.327; 0.022<br>Adjusted: 0.302; 0.037
ΔFM - ΔHOMA-IR	Crude: 0.436; 0.017<br>Adjusted: 0.390; 0.020
ΔFMI -ΔInsulin	Crude: 0.341; 0.017<br>Adjusted: 0.316; 0.030
ΔFMI - ΔHOMA-IR	Crude: 0.461; 0.004<br>Adjusted: 0.417; 0.013

ΔVF - ΔCRP	Crude: 0.305; 0.035<br>Adjusted: 0.308; 0.035
ΔThigh SAT -ΔInsulin	Crude: 0.349; 0.025<br>Adjusted: 0.324; 0.035
ΔtASAT - ΔHDL-cholesterol	Crude: -0.447; 0.002<br>Adjusted: -0.444; 0.002
ΔtASAT - ΔTriglycerides	Crude: 0.381; 0.008<br>Adjusted: 0.382; 0.008
ΔsASAT - ΔHDL-cholesterol	Crude: -0.398; 0.006<br>Adjusted: -0.395; 0.005
ΔsASAT - ΔTriglycerides	Crude: 0.405; 0.004<br>Adjusted: 0.405; 0.005
ΔsASAT - ΔIL-6	Crude: 0.340; 0.024<br>Adjusted: 0.340; 0.026
ΔPreperitonealAT -ΔVitamin D	Crude: -0.298; 0.046<br>Adjusted: -0.299; 0.049
ΔFFMI -ΔHOMA-IR	Crude: -0.333; 0.044<br>Adjusted: -0.328; 0.042
ΔSMMI - ΔTotal-cholesterol	Crude: -0.261; 0.052<br>Adjusted: -0.255; 0.053
ΔSMMI - Δnon-HDL-cholesterol	Crude: -0.260; 0.055<br>Adjusted: -0.260; 0.053
ΔASMMI -ΔHDL-cholesterol	Crude: -0.279; 0.039<br>Adjusted: -0.263; 0.053
ΔASMMI -ΔVitamin D	Crude: -0.303; 0.027<br>Adjusted: -0.309; 0.026
Δ RF thickness - ΔMyostatin	Crude: -0.288; 0.084<br>Adjusted: -0.288; 0.087
ΔFM/FFM ratio -ΔInsulin	Crude: 0.358; 0.012<br>Adjusted: 0.335; 0.020
ΔFM/FFM ratio -ΔHOMA-IR	Crude: 0.524; 0.001<br>Adjusted: 0.487; 0.003
ΔSMM/weight - ΔHbA1c	Crude: 0.414; 0.004<br>Adjusted: 0.477; 0.001
ΔSMM/weight - ΔHOMA-IR	Crude: -0.335; 0.042<br>Adjusted: -0.286; 0.089
ΔSMM/weight -ΔTotal-cholesterol	Crude: -0.268; 0.046<br>Adjusted: -0.326; 0.014
ΔASMM/BMI -ΔHDL-cholesterol	Crude: 0.274; 0.043<br>Adjusted: 0.253; 0.065
ΔASMM/BMI -Δnon-HDL-cholesterol	Crude: -0.275; 0.042<br>Adjusted: -0.316; 0.019
ΔASMM/BMI -ΔCRP	Crude: -0.352; 0.011<br>Adjusted: -0.344; 0.015

Δ%Weight -Δ%HDL-cholesterol	Crude: -0.338; 0.012<br>Adjusted: -0.319; 0.019
Δ%Weight - Δ%Vitamin D	Crude: -0.373; 0.006<br>Adjusted: -0.389; 0.005
Δ%Weight - Δ%Myostatin	Crude: -0.335; 0.033<br>Adjusted: -0.321; 0.041
Δ%BMI - Δ%HDL-cholesterol	Crude: -0.330; 0.014<br>Adjusted: -0.310; 0.022
Δ%BMI - Δ% Triglycerides	Crude: 0.268; 0.046<br>Adjusted: 0.290; 0.034
Δ%BMI - Δ%Vitamin D	Crude: -0.330; 0.016<br>Adjusted: -0.344; 0.014
Δ%BMI - Δ%Myostatin	Crude: -0.355; 0.023<br>Adjusted: -0.342; 0.029
Δ%WC - Δ%IL-6	Crude: -0.326; 0.019<br>Adjusted: -0.325; 0.022
Δ%WC/Ht - Δ%IL-6	Crude: -0.326; 0.019<br>Adjusted: -0.325; 0.022
Δ%Xc - Δ% Triglycerides	Crude: 0.284; 0.034<br>Adjusted: 0.284; 0.035
Δ%PhA/BMI - Δ%HbA1c	Crude: 0.397; 0.006<br>Adjusted: 0.427; 0.003
Δ%FM_% - Δ%HbA1c	Crude: -0.324; 0.026<br>Adjusted: -0.450; 0.003
Δ%FM_% - Δ%Insulin	Crude: 0.278; 0.053<br>Adjusted: 0.241; 0.104
Δ%FM_% -Δ%HOMA	Crude: 0.452; 0.005<br>Adjusted: 0.400; 0.020
Δ%FM_% -Δ%HDL-cholesterol	Crude: -0.282; 0.037<br>Adjusted: -0.256; 0.069
Δ%FM_kg - Δ%HbA1c	Crude: -0.295; 0.044<br>Adjusted: -0.391; 0.010
Δ%FM_kg -Δ%HOMA	Crude: 0.362; 0.028<br>Adjusted: 0.301; 0.077
Δ%FM_kg -Δ%HDL-cholesterol	Crude: -0.288; 0.033<br>Adjusted: -0.264; 0.057
Δ%FM_kg - Δ%CRP	Crude: 0.306; 0.029<br>Adjusted: 0.297; 0.039
Δ%FMI -Δ%HOMA	Crude: 0.430; 0.008<br>Adjusted: 0.375; 0.026
Δ%FMI -Δ%HDL-cholesterol	Crude: -0.273; 0.044<br>Adjusted: -0.246; 0.078
Δ%FMI - Δ%CRP	Crude: 0.237; 0.094

	Adjusted: 0.226; 0.120
Δ%Thigh SAT -Δ%Insulin	Crude: 0.362; 0.020<br>Adjusted: 0.346; 0.022
Δ%tASAT - Δ%HDL-cholesterol	Crude: -0.460; 0.001<br>Adjusted: -0.456; 0.001
Δ%tASAT - Δ% Triglycerides	Crude: 0.329; 0.023<br>Adjusted: 0.329; 0.024
Δ%sASAT - Δ%HDL-cholesterol	Crude: -0.410; 0.004<br>Adjusted: -0.408; 0.004
Δ%sASAT - Δ% Triglycerides	Crude: 0.368; 0.010<br>Adjusted: 0.368; 0.011
Δ%PreperitonealAT - Δ%Vitamin D	Crude: -0.293; 0.051<br>Adjusted: -0.297; 0.052
Δ%FFMI - Δ%HOMA	Crude: -0.351; 0.033<br>Adjusted: -0.343; 0.032
Δ%ASMMI -Δ%HDL-cholesterol	Crude: -0.278; 0.040<br>Adjusted: -0.263; 0.053
Δ%ASMMI -Δ%Vitamin D	Crude: -0.339; 0.013<br>Adjusted: -0.345; 0.013
Δ%RF-CSA - Δ%CRP	Crude: -0.325; 0.036<br>Adjusted: -0.316; 0.045
Δ%RF-CSA -Δ%Vitamin D	Crude: 0.309; 0.041<br>Adjusted: 0.317; 0.038
Δ%SMM/weight - Δ%HbA1c	Crude: 0.460; 0.001<br>Adjusted: 0.513; <0.001
Δ%SMM/weight - Δ%HOMA	Crude: -0.372; 0.023<br>Adjusted: -0.327; 0.046
Δ%ASMM/BMI -Δ%HbA1c	Crude: 0.315; 0.031<br>Adjusted: 0.379; 0.011
Δ%ASMM/BMI -Δ%HDL-cholesterol	Crude: 0.284; 0.035<br>Adjusted: 0.265; 0.052
Δ%ASMM/BMI -Δ%non-HDL-cholesterol	Crude: -0.264; 0.051<br>Adjusted: -0.298; 0.028
Δ%ASMM/BMI -Δ%CRP	Crude: -0.340; 0.015<br>Adjusted: -0.334; 0.018

The variables were rank transformed to be included in the models.ASMM: Appendicular Skeletal Muscle Mass; ASMMI: Appendicular Skeletal Muscle Mass Index; AT: Adipose Tissue; BMI: Body Mass Index; CRP: C-reactive protein; ECW: Extracellular Water; FFM: Fat-Free Mass; FFMI: Fat-Free Mass Index; FM: Fat Mass; FMI: Fat Mass Index; HbA1c: glycosylated haemoglobin; HDL: High-density lipoprotein; HOMA-IR: Insulin Resistance Homeostatic Model Assessment; IL-6: Interleukin 6; PhA: Phase Angle; RF: rectus femoris; RF-CSA: rectus femoris cross-sectional area; SAT: subcutaneous adipose tissue; sASAT: superficial abdominal subcutaneous adipose tissue;

SMM: Skeletal Muscle Mass; SMMI: Skeletal Muscle Mass index; tASAT: total abdominal subcutaneous adipose tissue; TBW: Total Body Water; VF: Visceral Fat; WC: Waist Circumference; WHtR: Waist–to–Height Ratio; Xc: Reactance.

Supplementary table S4: Post-hoc power analysis for not-significant changes after one year of follow-up

	n	Cohen's *d* for paired samples	Power obtained
WC (cm)	61	0.23	0.424
WHtR (cm/cm)	61	0.22	0.394
Rz (Ohm)	61	0.14	0.190
FFMI (kg/m2)	61	0.03	0.056
ASMMI (kg/m2)	61	0.11	0.135
VF (L)	58	0.06	0.073
HGS (kg)	61	0.31	0.664
sASAT (cm)	53	0.23	0.376
Glycemia (mg/dL)	60	0.02	0.053
HbA1c (%)	47	0.23	0.339
Insulin (µUI/mL)	49	0.14	0.161
HOMA-IR	37	0.10	0.091
Total cholesterol (mg/dL)	56	0.22	0.366
HDL-cholesterol (mg/dL)	55	0.13	0.157
Non-HDL-cholesterol (mg/dL)	55	0.19	0.283
LDL-cholesterol (mg/dL)	55	0.22	0.361
Triglycerides (mg/dL)	56	0.05	0.066
CRP (mg/L)	51	0.21	0.313
IL-6 (pg/mL)	51	0.06	0.070
Vitamin D (ng/mL)	53	0.23	0.376
Myostatin (ng/mL)	41	~0.00	0.050
FGF-21 (pg/mL)	40	0.22	0,274

ASMMI: Appendicular Skeletal Muscle Mass Index; CRP: C-reactive protein; FGF-21: Fibroblast growth factor 21; FFMI: Fat-Free Mass Index; Glyc: Glycemia; HbA1c: glycosylated haemoglobin; HDL: High-density lipoprotein; HGS: Handgrip strength; HOMA-IR: Insulin Resistance Homeostatic Model Assessment; IL-6: Interleukin 6; LDL: Low density lipoproteins; Rz: Resistance; sASAT: superficial abdominal subcutaneous adipose tissue; VF: Visceral Fat; WC: Waist Cir-cumference; WHtR: Waist–to–Height Ratio.

Cohen's d: difference between two means divided by the pooled standard deviation (effect size: **0.2** = small effect; **0.5** = medium effect; **0.8** = large effect). Power was calculated with jPower Module from jamovi Cloud (https://cloud.jamovi.org/), assuming a two-sided criterion for detection that allows for a maximum Type I error rate of α = 0.05.

This table presents the results of post hoc power analyses conducted for variables in which changes after one year of follow-up were not statistically significant. For each variable, the observed effect size, sample size, and corresponding statistical power are listed to provide context regarding the study's ability to detect meaningful differences over time. These analyses help clarify that the non-significant findings may be due to insufficient statistical power.
